# A Systematic Review of Home-Setting Psychoeducation Interventions for Behavioral Changes in Dementia: Some Lessons for the COVID-19 Pandemic and Post-Pandemic Assistance

**DOI:** 10.3389/fpsyt.2020.577871

**Published:** 2020-09-29

**Authors:** Gilberto Sousa Alves, Maria Eduarda Casali, André Barciela Veras, Carolina Gomes Carrilho, Eriko Bruno Costa, Valeska Marinho Rodrigues, Marcia Cristina Nascimento Dourado

**Affiliations:** ^1^ Translational Psychiatry Research Group, Federal University of Maranhão, São Luís, Brazil; ^2^ Post Graduation in Psychiatry and Mental Health (PROPSAM), Institute of Psychiatry, UFRJ, Rio de Janeiro, Brazil; ^3^ Dom Bosco Catholic University (UCDB), Campo Grande, Brazil; ^4^ Medical School, State University of Mato Grosso do Sul (UEMS), Dourados, Brazil; ^5^ Center for Alzheimer’s Disease, Institute of Psychiatry, Universidade Federal do Rio de Janeiro (UFRJ), Rio de Janeiro, Brazil

**Keywords:** dementia, psychoeducation, psychosocial intervention, caregiver, COVID-19

## Abstract

**Background:**

Impacts of social isolation measures imposed by COVID-19 Pandemic on mental health and quality of life of older adults living with dementia and their caregivers remain unexplored. Studies have shown that psychoeducational and psychosocial interventions can manage behavioral and psychological symptoms in dementia (BPSD) and reduce the emotional burden on family members when applied in home-setting scenarios.

**Method:**

a comprehensive systematic review of useful interventions for easing the BPSD burden in patients with dementia (PwD) and their caregivers in the context of COVID-19 quarantine was performed from January 2010 to March 2020.

**Results:**

From a total of 187 articles retrieved from electronic databases (MEDLINE, LILACS, Cochrane and SCOPUS), 43 studies were eligible for this review. Most of the psychosocial and psychoeducational interventions described were person-centered strategies based on the cognitive-behavioral approach or informational tools to enhance care providers’ knowledge of dementia. Most studies achieved successful results in handling BPSD and mood-anxiety symptoms of care providers, contributing to an overall improvement in dyad life quality.

**Conclusion:**

Evidence from the last few years suggest that low-cost techniques, tailored to the dyad well-being, with increasing use of technology through friendly online platforms and application robots, can be an alternative to conventional assistance during COVID-19 Pandemic. Nevertheless, the world’s current experience regarding the duration of the COVID-19 Pandemic and its effects on the cognition, behavior, and life quality of PwD will demand research on preventive and protective factors of dementia and the pursue of efficient interventions in different scenarios.

## Introduction

Dementia is a progressive syndrome and the associated functional decline inevitably leads to increasing dependence on others in different activities of daily living (1, 2). Patients with dementia (PwD) represent a heterogeneous group regarding diagnosis, stages of the disease, and level of functional decline. Behavioral and psychological symptoms in dementia (BPSD) are present at some point in almost 90% of PwD (3, 4), which is related to increased morbidity and mortality, caregiver burden, early institutionalization, and reduced survival (5, 6). One recent estimation of the global costs of dementia in Brazil cited values of US$1,012.35 (7). Although the economic and social impact remains to be further understood, the elevated healthcare costs resulting from the use of higher doses of psychoactive medications and falls, treatment dropouts, and wandering have all been thoroughly described in the literature (8).

A large number of PwD, mostly in moderate and advanced stages, require constant supervision (9). Since they are at the forefront of care, caregivers—family members and professionals, have a strategic role in the PwD quality of life and survival. A model of coping with stress is embedded in multiple stress-based problems, such as lack of social interactions, financial difficulties, frustration, anxiety, reduction of leisure activities, and concerns about the future (10, 11). Therefore, caregivers need professional assistance to cope with dementia, as they are a group particularly vulnerable to emotional burden, depression, and physical exhaustion. (10, 12).

Also, the COVID-19 Pandemic, which started in September of 2019 in Wuhan, the capital city of Hubei Province (China), had a crucial psychosocial impact on the mental health of older adults with pre-diagnosed dementia, especially after social isolation measures such as lock-down, and is still an unexplored topic. Despite dementia’s heterogeneity and psychoeducation measures, which are defined as a set of information provided by healthcare professionals that help in understanding the biological and social phenomena involved in the illness process and contribute to delivering higher-quality care in a home setting (13).

Measures directed to the dyad—caregiver and PwD—at home can be of a psychoeducational or psychosocial nature. Psychosocial interventions, defined as a set of techniques developed to use cognitive and behavioral mechanisms to promote the caregiver and PwD psychological well-being, can be associated with psychoeducation. Evidence shows that both measures, when aimed at understanding dementia and managing behavioral changes in a home setting and social isolation, can benefit PwD therapeutically, minimizing complications and reducing the emotional burden on family members during the isolation period, when social contact with specialized services is limited.

Furthermore, evidence has shown that PwD wish to participate in interventions that enhance their well-being, confidence, health, social participation, and human rights. This point highlights a need for improvements in psychosocial research to capture these outcomes (14). The present article aims to discuss psychoeducation measures and brief psychosocial interventions designed in a home setting, based on an integrative literature review, to manage behavioral changes in individuals with dementia and social isolation, which may be useful for the COVID-19 pandemic and post-pandemic assistance.

## Methods

An integrative literature review was conducted to gather and summarize the evidence available from original articles for the issue investigated. This integrative review study included six stages: 1 – formulation of the central research question (theme identification); Step 2 – definition of inclusion and exclusion criteria and literature search; Step 3 – categorization of primary studies (description of data to be extracted from the selected studies); Step 4 – assessment of the studies included; Step 5 – interpretation of results; 6 – knowledge synthesis of the results obtained from the studies assessed (15–17).

The central research question was formulated using the PVO method, where P is the study population (adults over 60 years of age with a diagnosis of dementia); V is the variable (psychoeducation measures), and O is the outcome (BPSD management).

Our review’s guiding question was: “Which psychoeducational and psychosocial measures are used for easing the BPSD burden in both PwD and their caregivers in the home setting?” The inclusion criteria were English language articles in the electronic databases (Medical Literature Analysis and Retrieval System Online (MEDLINE), Latin American and Caribbean Health Sciences Literature (LILACS), Cochrane, and SCOPUS); cross-sectional or prospective design; outpatient or population-based samples of adults over 60 years of age with irreversible and progressive dementia (e.g., Alzheimer’s disease, vascular dementia; Parkinson’s dementia); and non-pharmacological treatment through psychoeducation and psychosocial measures.** **The exclusion criteria were guidelines, systematic reviews, institutional protocols, psychoeducation measurements in other psychiatric or neurological conditions, and psychosocial intervention in hospitalized patients. The publications were individually searched and selected by two investigators during March and April 2020 and included papers from January 2010–April 2020.

The Preferred Reporting Items for Systematic Review and Meta-Analyses (PRISMA) guidelines (18) were used as a basis for the search and selection of studies (**Supplement Material**). A search strategy was created to conduct searches in the following databases: MEDLINE *via* PubMed from the US National Library of Medicine, LILACS, Cochrane, and SCOPUS with no time restriction. To expand our search, we chose to use a natural controlled language. The following descriptors (bold), synonyms, natural language, and Boolean operators were used to cross-check the databases: MEDLINE (Medical Subject Headings [MeSH]: search strategy – (aged or elderly or old or elder) and (non-pharmacological treatment or psychosocial treatment or ”) and (“Alzheimer disease” or Alzheimer’s) and (“dementia” or “cognitive dysfunction”).

Two investigators independently conducted the literature search and data extraction to minimize selection bias (misinterpretation of results and study design), and any discrepancies were resolved by consensus. We also performed a qualitative rating (see **Supplement Material**) of all selected studies through the Newcastle-Ottawa-Scale score (http://www.ohri.ca/programs/clinical_epidemiology/nosgen.pdf).

## Results

The study selection process, according to the PRISMA guidelines, is illustrated in **Supplement Material** flow chart. A total of 187 articles were retrieved and read, and from these, only 43 studies were considered eligible for our review. The selected studies are described in **Tables 1**, **2**. The sample sizes ranged from 6 to 555 participants. They were conducted in 16 countries, with the most substantial proportion nested in Europe (n = 23, 53.49%) and United States (n = 10, 23.26%). In contrast, the same ratio (n = 9, 20.93%) was found in Eastern Asia or Oceania, and only one was in Africa (2.32%). No studies were found for Latin America.

**Table 1:** Characteristics of Intervention studies with patients with dementia and caregivers*.

Authors, Year	CountryStudy Design	Outcome measures	Sample Size-Mean Age	Intervention Tool used	Main Results
Arritxabal et al. (1)	Spain Interventional	To evaluate a psychoeducational intervention program centered on the regulation of the emotion among caregivers.	Informal caregivers Intervention: n = 52 (56 ± 13) Control group: n = 32, 54.10 ± 12.30)	Cuestionario de Pensamientos Disfuncionales sobre el Cuidado, CBI, PSQ, PANAS, SWLS, TMMS-24, CES-D, PSS	When compared with the control condition, the experimental group obtained higher scores in positive affect, (subjective well-being, regulation of emotions, and satisfaction with caregiving), but obtained lower values in perceived stress and negative affect. The experimental group showed a significant decrease in dysfunctional thoughts and emotional attention. The control group registered higher levels of psychosocial support and lower satisfaction with caregiving
Boersma et al. (2)	The Netherlands Multiple case study	To perform a process analysis of the implementation of the Veder contact method.	Caregivers: n = 42 (47 ± 10.02)	Focus groups and interview	The reach of the intervention and aspects of implementation-effectiveness facilitated implementation. Both facilitators and barriers were identified. Little effort was put into maintenance: only one nursing home developed a long-term implementation strategy.
Chen et al. (3)	China Randomized controlled trial	To develop an intervention targeted towards improving coping strategies and reducing caregiver burden	Caregivers: n = 46 Intervention group: n = 24 (54.8 ± 15.1), Controls: n = 22 (55.1 ± 11.1)	RMBPC, CBI Chinese version, WCCL-R	Individual psychosocial and education interventions can be beneficial in terms of reducing the caregiver burden.
Clarke et al. (4)	England Randomized controlled	To develop a group-based adapted mindfulness program for people with mild to moderate dementia in care homes	Total = 31 caregivers Group intervention: n = 20 (81.30 ± 9.29) Controls: n = 11 (79.36 ± 9.91) and 28 participants post-test.	MBI manual, CSDD, RAID, QLAD, MMSE, PSS-13, MBAS	There were no significant changes between groups in terms of depression or anxiety.
Dam et al. (5)	The Netherlands Randomized controlled trial	To evaluate the effects of Inlife and its effectiveness and feasibility for caregivers of PwD	Total = 122 caregivers (> 18 years)	PPQ, SSCQ, MSPSS, SSL12-I, LS, LSNS-6, HADS, ICECAP-O, CarerQol, PSS, CRA	The study provided insights into the usability and implementation of online social support interventions in dementia care.
Fossey et al. (6)	United Kingdom Cluster‐randomized controlled trial	To use WHELD, or not, in the psychosocial approach for PwD	Total = 47 care home staff within nine care homes in the United Kingdom	WHELD program	Participants attributed effectiveness in using the WHELD approach to both patients and caregivers.
Gaugler et al. (7)	United States Longitudinal	To estimate the effects of comprehensive psychosocial support on spouse caregivers’ well-being trajectories related to the nursing home placement transition.	Total = 406 spouse caregivers of community-dwelling persons with Alzheimer’s disease Treatment: n = 203 71.55 ± 8.7 Usual care: n = 203 71.03 ± 9.5	ZBI, GDS, Global Deterioration Scale	Longitudinal models found that wives were more likely than husbands to indicate reductions in burden in the months after placement in an institution. Wives also reported more significant decreases in depressive symptoms after placement in an institution when compared with husbands.
Guzmán et al. (8)	United Kingdom Follow up	Monitor individual behavior and mood diaries through DMAS-17	Total: 10 PwD from two care homes and one nursing home Age interval 78–95 years Education: 9–12 years MMSE: 14–26	12-week Dance therapy sessions	A small to medium change was seen in behavioral and mood items, such as energy levels to socialize, increased appetite, mobility, and decreased irritability and depressed appearance.
Jones et al. (9)	Australia Cluster-randomized controlled trial	To explore whether the severity of cognitive impairment and agitation of older PwD predict outcomes in engagement, mood states, and agitation after an intervention with the robotic seal, PARO	N = 138 caregivers (intervention group) Age 84 ± 8.4	Robotic seal PARO, CMAI-SF, RUDAS	In clinical practice, PARO should be restricted to people with low-moderate severity of agitation.
Karel et al. (10)	Germany Longitudinal Multicomponent training program	To evaluate the feasibility and effectiveness of STAR-VA, an interdisciplinary program to manage behavioral disturbances in nursing-home residents with dementia	Total: 17 community living centers, PwD veterans > 60 years old (n = 71) Caregivers > 18 years old	STAR-VA training program Functional assessment staging tool Cornell Scale for Depression in Dementia Rating Anxiety in Dementia Scale Cohen-Mansfield Agitation Inventory	Behaviors were clustered into six domains: care resistance, agitation, aggression, vocalization, wandering and others. Frequency and severity of agitation and mood symptoms decreased with effect sizes ≥1
Kerssens et al. (11)	United States Longitudinal	To test the usability, feasibility and adoption of the Companion in a home- and community-based setting	Total = 7 dyads of PwD and caregivers PwD: median age 77 (60–88) Caregivers: median age 79 (63–86)	Barthel Index, MMSE, Lawton, CSDD, NPI, CSI, ZARIT	The technology was easy to use, significantly facilitated meaningful and positive engagement and simplified caregivers’ daily lives. Caregivers had high expectations of their loved one’s ability to regain independence. Care recipients used the system independently but were limited by cognitive and physical impairments.
Matsuzomo et al. (12)	Japan Follow-up Cluster randomized	To investigate the effects of BASE on challenging behavior of home‐dwelling PwD	Home caregivers (n = 24) Professionals (n = 49) Controls: n = 70 (84.9 ± 6.7) PwD: n = 141 (83.7 ± 7.1)	BASE program	Significant reduction in BPSD in the intervention group after 6 months as compared with the CG (11.6 to 10.8; P <.05).
Nakanishi et al. (13)	Japan Cluster‐randomized controlled trial	To investigate the effect of the BASE program on challenging behavior in home-dwelling PwD	Total = 283 PwD Intervention group: n = 141 Control group: n = 142 Total: n = 95 care professionals Intervention group: n = 46 Control group: n = 49	BASE program	Significantly less challenging behavior in the intervention group compared to the control group follow-up.
Nakanishi et al. (14)	Japan Cluster-randomized controlled study	To identify a key component of the psychosocial dementia care program that is associated with a reduction in challenging behavior	Total: 305 participants PwD: n = 219 (83.8 ± 6.9) Care professionals: n = 86 (45.6 ± 5.3)	NPI-NH, Abbey pain Scale Japanese version, SMQ JV, Barthel Index JV, ATC, SCIDS JV	There was a significant reduction both in challenging behavior and pain from baseline to the follow-up assessment.
Stockwell-Smith et al. (19)	Australia Explanatory sequential mixed-method design	To evaluate the effect of a targeted community-based psychosocial intervention	Total: 88 dyads Care recipient n = 45 > 65 years	Early Diagnosis of Dyadic Intervention	There were no significant between-group differences in either Symptom Management and Support Service self-efficacy.
Van Mierlo et al. (20)	The Netherlands Cluster randomized controlled trial	To evaluate the effectiveness of DEM-DISC on informal caregivers and people with dementia. To investigate its user-friendliness and usefulness among informal caregivers of people with dementia and case managers who provide care coordination and continuity of care in community-dwelling people with dementia. To investigated which facilitating and impeding factors were expected to influence the further nationwide implementation of DEM-DISC.	Total: 73 informal caregivers, 19 randomized case managers, and 41 professional caregivers Experimental group: n = 54 63.0 ± 11.6 Control group: n = 46 60.4 ± 12.7)	MMSE, MDS-care receiver, CANE, Qol-AD, NPI, SSCQ, USE	Informal caregivers who used DEM-DISC for twelve months reported an increased sense of competence than controls. A subgroup of users who frequently accessed DEM-DISC reported more met needs after six months than controls. Overall informal caregivers and case managers judged DEM-DISC as easy to learn and user-friendly.
Williams et al. (21)	United States Controlled clinical trial	To determine whether video-based coping skills (VCS) training with telephone coaching reduces psychosocial and biological markers of distress in primary caregivers	116 primary caregivers Intervention group: n = 59 (62.1 ± 13.6) Waiting list: n = 57 (59.0 ± 12.8)	PSS, STAI, STAXI, CES-D, MMPI, CGSE, PSQI, Blood pressure (BP) and heart rate (HR) were recorded during stress testing using an automatic blood pressure monitor, Salivettes^®^,	The group main effect was significant for depressive symptoms and perceived stress, and there was a trend for the group effect for trait anxiety. None of the Group × Visit interactions were significant. A Group × Visit interaction was found for both mean systolic and mean diastolic blood pressure, with a significant group main effect for mean diastolic blood pressure maintained over the six-month follow-up period.

*Tables 1 and 2: references are available at the **Supplementary Material**.

**Table 2:** Characteristics of Intervention studies with patients with dementia and caregivers*.

Authors, Year	CountryStudy Design	Outcome measures	Sample SizeMean age (SD)	Intervention Tool used	Main Results
Bartels et al. (15)	The Netherlands A single-blinded randomized controlled trial	To examine the sustainability of positive intervention effects of the mobile health intervention on caregivers’ well-being	Total: n = 76 caregivers (72.1 ± 8.4) Experimental group: n = 26 (71.7 ± 8.4) Pseudo-experimental: n = 24 (71.1 ± 7.3) Control group: n = 26 (73.2 ± 9.4)	SSCQ, PSS, PMS, CES-D, HADS-A, NPI-Q, CDR	The results obtained showed that the intervention “Partner in Sight” can reduce feelings of stress, depressive symptoms and enhance a sense of competence in caregivers.
Brown et al. (16)	United States Randomized controlled trial	To test the efficacy of MBSR program for reducing caregiver stress and enhancing the care giver-recipient relationship	Total: 38 caregivers (MBSR group n = 23, SS group n = 15). Age of participants: 61.14 ± 10.41 (39–88 years)	MBSR program	Caregiver participants in MBSR reported lower levels of stress, tension and anger. The SS intervention highlighted an understanding and acceptance of dementia behaviors, which can help to reduce the perceived burden.
Bruvik et al. (17)	Norway Assessor-blinded multicenter RCT	To describe a multicomponent tailored psychosocial intervention trial design to reduce depressive symptoms in PWD and caregivers	Total: 230 dyads of home-dwelling PWD and a principle family caregiver Intervention group (n = 115): caregiver 64.1 ± 12.2, PwD 78.3 ± 7.5; Control group (n = 115): caregiver 62.9 ± 11.4, PwD 78.5 ± 7.5	CSDD, GDS, RRS Norwegian version, MMSE NV, NPI-Q, PSMS, IADL	The study did not find that a structured, multicomponent and tailored psychosocial intervention program significantly reduced depressive symptoms in PWD or their family caregivers compared to usual care.
Burns et al. (18)	USA, Australia and the UK Randomized controlled trial	To assess whether caregiver interventions can still be successful when anti-dementia drugs are provided to patients	158 dyads divided equally across three centers: Sydney (n = 52), New York (n = 52) and Manchester (n = 54). Sydney: Patients 75.0 mean age (58–89 years), caregivers 71.8 (53–86); Manchester: Patients 72.7 (52–91), caregivers 72.2 (49–88); New York: Patients 73.6 (55–89), caregivers 70.2 (47–88).	MMSE, GDS, BAI, RMBPC, BDI, Stokes Social Network List, WFCS, PMS, EuroQol,	The caregiver intervention was associated with positive results on caregiver depression across all the countries.
Dahlrup et al. (22)	Sweden A quasi-experimental longitudinal cohort study	To examine the effects of a psychosocial intervention for family caregivers in describing symptoms of dementia	Intervention group: n = 129 (61 ± 12.9) Control group: n = 133 (62 ± 12.6) PWD: n = 144 (85 ± 5.9);	MMSE, GBS-scale, The Berger scale, IADL	The family caregivers who underwent psychosocial intervention achieved a better understanding of different symptoms and the behaviors of dementia.
Davis et al. (23)	United States Randomised controlled trial	To study the preliminary efficacy of a telephone intervention (FITT-NH) for improving dementia caregivers’ adjustment	Total: 27 caregivers assigned to FITT-NH and 26 to the non-contact control condition. Caregivers in the intervention group: 57.25 ± 10.67 Care recipient: 82.54 ± 5.48 Caregivers in the control group: 61.32 ± 10.46 Care recipient: 82.73 ± 9.05	FITT-NH	Caregivers receiving FITT-NH showed reduced guilt feelings and more staff positive interactions compared to those caregivers with no additional contact.
Den IJssel et al. (24)	The Netherlands Cluster randomized controlled trial	To evaluate the effect of the intervention on nursing staff burnout, job satisfaction, and job demands.	nursing staff: n = 305 (43.5 years ± 12.2)	APID, NPI-Q, CANE Dutch version, UBOS DV, Leiden Quality of Work Questionnaire	The intervention showed no additional improvement in three dimensions of burnout, job satisfaction and job demands.
Gaugler et al. (25)	United States A single-blinded randomized controlled trial	To evaluate the effects of NYUCI-AC on decreases in family and role conflict and increases in perceived social support	Total n = 107 (treatment group n = 54 and control group n = 53). Total: 50.46 ± 8.24 Control: 49.68 ± 9.36 Treatment: 51.23 ± 6.95	NYUCI-AC	Effectiveness in reducing residential long term care placement for persons with ADRD and adult child caregivers’ adverse reactions to disruptive behavior problems, and depressive symptoms.
Johannessen et al. (26)	Norway Randomized controlled trial	To investigate the outcome of the study from the perspective of the healthcare professionals	19 health professionals 34–61 years	Psychoeducation of dementia and the management of its symptoms.	The intervention can prevent burnout of the primary caregivers and social isolation and thereby promote health.
Johannessen et al. (27)	Norway Randomized controlled trial	To investigate family caregivers’ experiences of a multicomponent psychosocial intervention program	20 family caregivers 50–82 years	Individual qualitative interviews and a psychosocial intervention program	It contributed to reducing the burden and loneliness caused by the disorder.
Koivisto et al. (28)	Finland Randomized controlled trial	To assess the influence of the intervention on AD progression, behavioral symptoms, and HRQoL	236 dyads of home-dwelling persons with AD and their family caregivers (control group n = 152; intervention group n = 84)	CDR-SOB, CERAD-NB, MMSE, ADCS-ADL, NPI, QoL-AD, VAS, BDI, SOC, 12-GHQ, 15D	The present study did not show any long-term effect of the early psychosocial intervention.
Langhammer et al. (29)	Norway Exploratory design	To evaluate whether a combined intervention of physical activity and music therapy could reduce anxiety, restlessness, irritability, and aggression	6 individuals with dementia and signs of frontal lobe problems PwD: n = 6 (75.6 ± 6.52) Caregiver: n = 6 (65.6 years ± 11.9) Mean age of 84.3 years	BVC, NPI-Q, Semi-structured interviews	Implementation of individualized music therapy combined with increased physical activity for eight weeks was a feasible intervention that reduced anxiety, restlessness, irritability, and aggression in the current study.
Liang et al. (30)	New Zealand Pilot block randomized controlled trial	To investigate the affective, social, behavioral, and physiological effects of the companion robot Paro for PwD	30 dyads (PwD and caregivers) PwD age range: 67–98 years Caregivers age range: 30–86 years	Paro	Paro helped improve mood, reduce anxiety, acting as a social stimulus, and increasing communication and cooperation with therapists and staff.
Lord et al. (31)	United Kingdom Randomized controlled trial	To evaluate the dissemination of the program Strategies for Relatives (START)	134 clinical psychologists and 39 admiral nurses -	START, individual interview	The study began the new intervention dissemination process.
McCurry et al. (32)	United States A randomized, controlled trial with blinded assessors	To test the effects of walking, light exposure, and a combination intervention (walking, light, and sleep education) on the sleep of persons with Alzheimer’s disease	132 AD participants and their caregivers Walking: 82.2 ± 8.50 Light: 80.6 ± 7.3 NITE-AD: 80.0 ± 8.2 Control: 81.2 ± 8.0	SDI, Actigraph, CSDD, SCQ, MMSE	To test the effects of walking, light exposure, and a combination intervention (walking, light, and sleep education) on the sleep of persons with Alzheimer’s disease
McCurry et al. (33)	United States Randomized controlled trial	To investigate the feasibility of implementing a Sleep Education Program (SEP) for improving sleep in an adult family home (AFH) residents with dementia, and the relative efficacy of SEP compared with usual care control	37 adult family home (AFH) staff-caregivers and 47 residents with co-morbid dementia and sleep disturbances. AFH staff-caregivers: 86.6 ± 7.2 Residents: 48.2 ± 9.7	Actigraphy, CSDD, RMBPC, ESS	To investigate the feasibility of implementing a Sleep Education Program (SEP) for improving sleep in an adult family home (AFH) residents with dementia, and the relative efficacy of SEP compared with usual care control
Moyle et al. (34)	New Zealand Randomized controlled trial	To compare a lifelike baby doll intervention for reducing agitation and aggression in older people with dementia in long-term care (LTC)	Total: 35 residents from five LTC facilities (Lifelike Doll n = 18, Usual Care n = 15). Total: 87.8 years ± 8.6 Intervention group: 86.1 ± 8.6 Control 89.7 (8.4)	Semi-structured interview, OERS, CMAI-SF, MMSE, NPI-NH	There was no statistical evidence to support the hypothesis that the lifelike baby doll intervention would reduce residents’ anxiety, agitation, and aggression.
Orrell et al. (35)	United Kingdom A single-blind pragmatic randomized controlled trial	To evaluate the effectiveness of a home-based, caregiver-led (iCST) program in (i) improving cognition and QoL for the PwD and (ii) mental and physical health for the caregiver.	A total of 356 dyads iCST group: n = 180 TAU group: n = 176		To evaluate the effectiveness of a home-based, caregiver-led (iCST) program in (i) improving cognition and QoL for the PwD and (ii) mental and physical health for the caregiver.
Pihet et al. (36)	Switzerland Quasi-experimental intervention that followed the TIDieR guidelines	To examine the feasibility and the effects of implementing the program and the participants’ use of the trained strategies	26 ICD through service providers in the field of dementia ICD median age of 68 years (*Q1* = 60, *Q3* = 72, *range* 37–86); Patients median age of 77 years (*Q1* = 71, *Q3* = 82, *range* 56–94)	Caregiver’s burden 22-items questionnaire, MBP, caregiver’s MBP-related distress, Ilfeld short version, VAS	The program resulted in substantial improvements in burden, psychological distress, self-efficacy and the increasing ICD quality of life.
Phung et al. (37)	Denmark Multicentre, randomized controlled rater-blinded trial	36-month follow-up to rate changes in behavioral symptoms and quality of life of both PwD and caregivers in 5 Danish districts	Counseling, psychosocial support; 163 patients to DAISY intervention group and 167 to control group	QoL-AD NPI ADCS-ADL GDS EQ-VAS	The 12-month follow-up study observed positive effects on preventing depressive symptoms and maintaining the quality of life among PwD. No effects were found on the caregiver’s quality of life after a 360-month follow-up.
Schall et al. (38)	Germany Randomized, wait-list controlled design	To relieve the sense of isolation experienced by many PwD, as well as the burden on family caregivers	44 PwD Intervention group n = 25, Wait-list control group n = 19). Intervention Group: 75.1 ± 7.70 Wait-list control group: 76.4 ± 8.68	ARTEMIS	ARTEMIS intervention provided positive effects on the emotional well-being and the self-assessment of quality of life in PwD and a reduction in apathy and depressive symptoms.
Shata et al. (39)	Egypt Randomized controlled trial	To develop and evaluate the efficacy of a multicomponent psychosocial intervention program for informal caregivers of persons with NCDs	114 patients (Intervention group n = 55 and control group n = 59) PWD: age range 61 -86 years: 69.29 ± 6.24 years. Total: 48.63 years (12.31); Intervention: 49.35 ± 11.89; Control: 47.97 ± 12.76	MMSE, Caregivers’ Dementia-related Knowledge Questionnaire, HDRS Arabic version, TMAS, ZBI, DRKQ	The study provided evidence for the short-term efficacy of a culturally sensitive multicomponent psychosocial intervention program in improving Dementia-related knowledge and the emotional status of informal caregivers of people with NCDs.
Søgaard et al. (40)	Denmark Randomized controlled trial	To investigate the impact of an early psychosocial intervention aimed at patients with Alzheimer’s disease (AD) and their caregivers	330 dyads Intervention group n = 163 and control group n = 167.	RUD	An AD intervention may burden the caregiver more than it saves costs in proper health care and institutionalization.
Søgaard et al. (41)	Denmark Randomized controlled trial	To assess the cost-utility of early psychosocial intervention for patients with Alzheimer’s disease and their caregivers.	Patients in the intervention group 76 years (**8**), caregivers 65 (13); Patients in the control group 75 (7), caregivers 66 (13) ≥50 years	RUD, EQ-5D, QALY	Psychosocial intervention is unlikely to be cost-effective in a Danish setting because it did not generate additional QALYs, and it led to the higher average usage of informal care.
Tremont et al. (42)	United States Randomized controlled trial	To examine the efficacy of the FITT-C to reduce depressive symptoms and burden in distressed dementia caregivers	250 dyads Caregivers – total sample: ± Intervention group: n = 133 (63.32 ± 12.30) Telephone support: n = 117 62.03 ± 13.75 PwD total sample: 78.06 ± 10.06, Intervention group: 79.22 ± 9.11 Control: 76.74 ± 10.93	FITT-C	The study demonstrated the equivalence of face-to-face and telephone assessments on two of the primary outcome measures (depressive symptoms, perceived burden, and reaction to memory and behavior problems).
Tremont et al. (43)	New England Randomize controlled trial	To examine the efficacy of Telephone Tracking-Dementia (FITT-D) and telephone support (TS) to promote psychoeducation, problem-solving, and a directive approach to behavioral disturbances.	≥50 years Intervention group: caregivers: 65.75 ± 13.71 Care recipient 75.94 ± 9.14; Control caregivers: 61.00 ± 9.60 PwD: 75.29 ± 10.79	FITT-D	Caregivers receiving the FITT-C used community support services more often than those receiving TS (P = .02). FITT-C caregivers had a significantly lower rate of emergency department visits (rate difference 9.5%, P = .048) and hospital stays (rate difference 11.4%, P = .01) over the 6-month course of the intervention than TS caregivers.

*Tables 1 and 2: references are available at the **Supplementary Material**.

Almost all studies (n = 42, 97.67%) employed psychosocial and-or psychoeducational strategies addressing the dyad, and only one used cognitive-behavioral intervention (2.33%) for the caregiver solely (**Table 1**). The majority of investigations (n = 33, 76.74%) were based on randomized controlled trials, as following: 1 interventional study design (2.22%), 1 multiple case (2.22%), 6 longitudinal studies (13.95%), 1 explanatory sequential mixed-method design (2.22%), 1 exploratory design (2.22%), 2 quasi-experimental interventions (4.44%), and 1 controlled clinical trial alternately assigned (2.22%). A few studies used more than one design method.

The studies included in our review evaluated participants with distinct levels of dementia, being most of them (n = 34, 79.07%) focused on mild-moderate dementia. In comparison, 8 (18.60%) other studies investigated mild Alzheimer’s disease, and only 1 (2.32%) moderate-severe dementia. Cognitive and functional scores were provided only by a few studies (n = 10, 23.26%). Most caregivers were familiar or informal caregivers (n = 30, 69.76%), albeit professional care providers could be found in the remaining studies (n = 13, 30.23%). For most of the compelled studies, the primary outcome result was evaluating and reducing behavioral disturbances in PwD, such as agitation, restlessness, anxiety (n = 17, 39.53%), including specific interventions for decreasing sleep disturbances (n = 2, 4.65%). Concerning caregivers, the most important outcome was the reduction of burden and stress (n = 21, 48.84%), including the attenuation of depression or other mood symptoms (n = 4, 9.30%). Also, studies aimed at evaluating caregiver wellbeing, quality of life and satisfaction of with caregiving (n = 6, 13.95%); some outcome measures comprised the enhancement of knowledge on dementia through psychoeducation and the development of a sense of competence in dealing with BPSD (n = 9, 20.93%) and reducing guilty and adverse reactions toward PwD (n = 2, 4.65%). Finally, other studies had primary goals evaluating online psychosocial support, including robot-guided psychosocial intervention (n = 3, 6.98%).

The primary interventions are summarized in the following topics.

### An Overview of the Psychosocial and Psychoeducational Interventions

Most of the studies of psychosocial and psychoeducational interventions for the field of dementia use an umbrella of techniques, most of them based on cognitive-behavioral therapy (CBT) (19) or a combination of psychotherapy and essential information on dementia (20). Caregivers were generally encouraged to share feelings about dementia such as guilt, loneliness, worry and sadness (20). The majority of studies carried out interventions for dyad (15). Caregivers may learn from CBT to develop self-monitoring of depressive or anxiety symptoms or help PwD do so (4). One study, for instance, employed CBT in PwD at moderate stages (16). The Coping with Caregiver model – CCM (5) articulates cognition and behavior with negative affective states and teaches cognitive-behavioral mood management skills. In one investigation consisting of a 14 h training program with CCM, the intervention group exhibited significantly less depressive symptoms and experienced lower caregiver burden than the control group at the end of study (5). The Residential Care Transition Module (RCTM) consists of a six-session, 4-month psychosocial intervention designed to help families manage their emotional and psychological distress following residential care placement of a cognitively impaired relative (17). Cognitive Stimulation Therapy (CST) is a psychosocial group intervention recommended by the UK NICE guidelines that have shown to improve cognition and quality of life (18). There is some evidence showing the efficacy of CST in apathy and depression-dysphoria (21). Multisensory stimulation (MS) comprises a set of sensory stimuli (visual, auditory, tactile) and controlled environment, following a schedule of reinforcement and has been studied in AD, Huntington’s disease (15). Mindfulness-based intervention (MBI) is based on paying attention in a particular way, i.e., at the present moment and non-judgmentally to enhance emotional regulation (22, 23). This meditation method focuses mainly on breath or body and open monitoring of the whole cognitive-affective field (22). One single-blind intervention conducted by Churcher Clarke and colleagues included a 10-session MBI with mild and moderate dementia and found a medium effect size improvement in overall quality of life, but no significant changes in depression or anxiety symptoms (22).

### Main Goals of Interventions

Overall, most psychoeducational and psychosocial interventions aimed to enhance care providers’ knowledge about the required skills of caregiving and, ultimately, to reduce dementia sufferers’ illness deterioration and institutionalization (24, 25). Most studies employed personalized and person-centered strategies (26, 27) designed to fulfill the needs, characteristics and preferences of both PwD and their caregivers (28). As an example, the Person-centered care (PCC), widely recognized concept in dementia research and care and the Dementia Care Mapping (DCM), a method for implementing PCC (29).

Some strategies seek to promote the general well-being and life quality of the dyad (17, 20), such as the case of the Dealing Well with Dementia project, which used the “Dignity Therapy” (30), the Family Intervention (FITT-C) (31) or the Northern Manhattan Caregiver Intervention Project, which addressed the relief of stress symptoms in Hispanic spouses of PwD in NYC (32). In Denmark, a large multicentric study (DAISY) evaluated the effectiveness of a program for outpatients with Alzheimer’s disease in 12 months (33, 34). The therapy was based on measures of education, counseling and support for family members (33). Raeanne and colleagues (35) evaluated the efficacy of the Pleasant Events Program (PEP), a 6-week Behavioral Activation intervention designed to reduce CVD risk and depressive symptoms in caregivers. According to the authors, the group receiving PEP intervention had significant reductions in depressive symptoms (*p* = .039) and negative affect (*p* = .021) from pre- to post-treatment (35).

Other examples included multiple activities, such as the Pleasant Events Program, in which a protocol comprised physical exercise, occupational therapy and support intervention for the dyad, have also been employed (28). The caregivers were encouraged to learn from cognitive stimulation through specific protocols, by dealing with their stress and anxiety feelings and the daily routine; this was the case of the individual Cognitive Stimulation Therapy (iCST) (36).

The promotion of well-being through dancing was also a therapeutic tool in some studies (37), improving social activity and psychical health. The Project DANCIN (Dance Therapy Intervention) measured in two daily sessions (in a total of 24 sessions) PwD with mild and moderate stages and caregivers who want to include this activity in their daily routine (38). Participants exhibited a set of BPSD assessed by the Dementia Mood Assessment Scale (DMAS-17) (39), including insomnia, agitation, angry outbursts, daytime drowsiness, continually fidgeting and staring at the floor and perseverative questioning (38). PwD and also those showing sensory or auditory deficits could benefit from the dancing sessions (38). Additionally, the absence of dance experience was not a limitation to overall engagement in the PwD group.

The reduction of psychological distress among familiar care providers was pursued by the START Project (Strategies for Relatives Intervention) by developing healthy coping strategies (40). Family member’s engagement was reinforced through partnership interventions, as a critical element to reduce behavioral disturbances and enhance well-being in another study (41).

The specific training of staff members showed useful outside metropolitan areas, where memory clinics are not available. One example is the Clinical Antipsychotic Trials of Intervention Effectiveness—CATIE-AD study (n = 421 AD outpatients), which implemented psychoeducation training for GP and non-specialists to the early identification of behavior disturbances, clarifying its main behavior dimensions. A total of 4 distinct clusters have been identified: a) agitation and irritability, b) apathy and eating problems, c) psychosis (delusions and hallucinations, and d) emotion and disinhibition (depression, euphoria and disinhibition) (42).

In other studies, self-monitoring skills were assessed both by the staff and the family member (43). In agitation management, a study aimed at satisfying basic needs proved effective in reducing verbal agitation (44). Improving the patient’s food intake and nutritional status is also essential to reduce agitation and improve this group’s quality of life (45).

### Adopting Tailored Activities

One aspect regarded as crucial to warranty the effectiveness of psychosocial interventions is the provision of tailored activities, particularly for home-dwelling PwD (9, 46). As the caregiver group usually varies from adolescents (including “adult children”), adults, spouses to professional care providers, individualized dyadic interventions shall be designed in any dyadic compositions to reduce the caregiver social strain (47) and improve PwD functional ability (47). One of the strategies credited as successful is the promotion of multiagency discussions, which enable the evaluation and provision of unmet needs (19). Dyadic interventions may also be addressed to the primary health care system (48), and GPs may receive training in psychosocial counseling (49). Another innovative intervention allowed personalized interventions to integrate home and residential care services in Japan (19). Noteworthy, the level of PwD engagement shall consider not only the degree of cognitive decline but the preservation of sensory stimuli (e.g., sight, smell, and touch), since potential sensory dysfunctions may be associated with apathy and isolation (23, 50).

Optimal care also involves adapting the expectations of both professionals and the dyad. In one interesting study conducted by Popham and colleagues (13), the main obstacles to optimal care through the Sheffield Care Environment Assessment Matrix (SCEAM) questionnaire (51). The main themes for the dyad were the lack of social interaction activities, more freedom for PwD to go outside, more freedom to choose what activities they could do according to the program, while health and safety, most of the times involving spatial restriction for the patient to wander, were the strong concern for health professionals (13). The support tool Inlife was launched in the Netherlands, developed explicitly for caregivers and PwD to lower the threshold for asking and support (52) in an ongoing 16-week RCT. Primary outcomes comprised the caregiver’s sense of competence and secondary, while secondary outcomes consist of evaluating mood symptoms (anxiety and depression), social network, and feelings of loneliness.

Albeit most studies showed successful results in stimulating PwD, negative results were also reported. One follow-up investigation of 3 years revealed no benefit on the well-being and delay of cognitive decline in mild and very-mild DA (53). The absence of regular weekly phone support and a lack of homogeneity in patient recruitment, including culturally heterogeneous groups, are significant limitations (53).

### Intervention Programs Targeting Home-Dwelling PwD and Caregivers

Evidence has suggested that caregivers living outside metropolitan areas (e.g., in rural areas) are more prone to develop emotional burden and instability in the dyad (54), mostly due to the scarcity of specialized facilities, including a memory outpatient service and the absence or lack of psychosocial counseling. Conversely, similarly to their counterparts in major cities, these subjects may benefit mainly from home-based psychosocial intervention targeting the caregiver’s depressive symptoms and burden (55) and short and long term complications associated with BPSD (56). Thus, both low cost and more comprehensive strategies should be favored, especially in times of pandemics. Some experiences have successfully engaged GPs in a psychosocial counseling initiative (41). The adaptation of the protocol “Living Well with Dementia” stimulated the search for psychosocial support among users of the United Kingdom’s primary healthcare system (21). One cluster-randomized trial conducted by Nakanishi and colleagues (19) in a local home setting implemented through a 6-month follow the BASE program, a palliative care approach lead by care managers and professional caregivers, which resulted in a significant reduction of challenging BPSD of PwD. The project Staff Training in Assisted Living Residences (STAR-VA) assessed the frequency and intensity of BPSD in veterans PwD in nursing home care (52). In Germany, the Project Future Workshop Dementia (Zukunftswerkstatt Demenz) has followed Family members and PwD in rural areas (57).

Home-based approaches, including a complete set of activities, such as cognitive and physical training combined, may exhibit better results in randomized controlled trials (RCTs) with community-dwelling PwD. The NYU Caregiver Intervention (NYUCI) was designed to provide caregiver support for adult children and prevent residential care placement through 2 years. The term “adult children” is applied to the child or teenager relatives, most of them sons or daughters or grandchildren compelled to assume caregiving duties, including personal hygiene, economy, and safety (58). NYUCI intervention included family counseling, support group referral and *ad hoc* consultation, or a contact control group. Participants of NYUCI were found to be less prone to admit their parents to a residential care setting (*p* < 0.05) and also delayed their parent’s time to admission significantly longer (228.36 days) than those of the control group (17).

### Interventions Based on Phone Calls and Internet Apps

Internet psychosocial interventions hold considerable promise for meeting the educational and support needs of informal dementia caregivers at reduced costs (52, 59). A number of them have been delivered to support caregivers (60). The types of intervention vary widely, as does the quality of the methods used (46). Person-centered care approaches designed to home settings have been performed using observational tools and practice development cycles, such as the Dementia Care Mapping™ (DCM™) (61). Besides, touch screen technologies, such as the Companion, have offered an exciting opportunity to deliver the psychosocial intervention and monitor BPSD and caregiver distress and represent a promising field of development for the caregiver network (62). The Dementia Digital Interactive Social Chart (DEM-DISC) is an e-advice ICT tool to support customized disease management in dementia. This study aimed to improve and evaluate DEM-DISC, its user-friendliness and usefulness, and investigate future implementation (63). A total of 73 informal caregivers of PwD, supported by 19 randomized case managers. This study demonstrates that using DEM-DISC positively affected the sense of competence and experienced (met) needs of informal caregivers (63). Care providers could also manifest their opinion about the user-friendliness and usefulness of DEM-DISC through telephone interviews.

The “Ability Program” conducted by Realdon and colleagues in RCT lasted six weeks and comprised cognitive, physical activities, and a set of devices measuring and monitoring remotely vital and psychical health parameters (64). Another relevant follow-up intervention was promoted by the FITT-C study, using telephone-based interventions with trained therapists to manage the caregiver´s depression and burden. Those who received the FITT-C along six months tended to seek less medical attention in the urgency and had fewer hospital stays than the control group (65).

## Discussion

Our review provided a concise perspective of the last ten years of research on psychoeducational and psychosocial interventions directed to PwD and caregivers. Most studies achieved successful results in handling BPSD and mood-anxiety symptoms of the care provider, leading to an increase in skills related to caring and contributing to an overall improvement of the dyad quality of life. Telephone-based interventions have also shown effectiveness in reducing presential medical consultation and hospitalization. Similarly, studies adapting to friend-technology devices, including robots and remote-monitoring apps, exhibited promising results for promoting knowledge and facilitating decision-making among care providers. The world currently experiences uncertainty on the COVID-19 pandemic duration, and its effects in the cognition, behavior, and quality of life of PwD are yet to be understood. The current review sheds light on this theme, highlighting the potential use of low-cost and high-impact strategies actionable at the home-dwelling during the quarantine and the post-pandemic period.

The existing approaches tend to favor elements of the dyadrelationship differently. Such aspects involve, in summary, caregivers’ awareness of what behavioral changes are. These approaches can range from simple monitoring to psychotherapy. Conversely, taking care of restlessness, apathy and other behavioral symptoms is also critical. If applied for the current pandemics, measures to monitor sleep, daily walks, and light exposure can counteract the prolonged quarantine period. Another critical aspect is promoting the caregiver’s well-being, by reducing depressive symptoms and burden related to the isolation and permanent contact with PwD. Feelings of being overwhelmed, frustration, and loss of family contact may benefit from regular support and assistance, as demonstrated by telephone-derived interventions (31, 65).

One exciting field of research, for instance, will be the home-based adaptation through technological devices of classic intervention tools, including visual arts (66), museum visitations (61), or artistic, educational workshops (67, 68). The overall adherence and engagement by caregivers to e-devices have shown to be enjoyable and positive (60, 63, 69). Furthermore, technology devices also offer an opportunity for disease management to health assistants (63). In the future, user-friendly ICT solutions may be used to promote self-management by informal caregivers and assist caregivers in finding appropriate care services tailored to their specific situation and needs. Albeit the benefits of computer-based assistive technology have been evidenced, barriers and impediments still threaten the extensive use of these tools, including the inability of partners and care providers to recognize its added value, the lack of potential financial investors and the lack of government support for the development and enhancement of such instruments (63). Possibly, the undetermined duration of pandemics will demand the need for modifying the current protocols and research programs through the emphasis of support group intervention (70) and optimal staff training (13). Future studies will also require personalized protocols to overcome regional challenges, such as the low access of material resources, diversity of school background and the profile of BPSD among PwD.

A multiplicity of factors in primary care may serve as obstacles to optimal primary dementia care, as pointed by previous studies (71), including challenges related to a) the complex biomedical, psychosocial, and ethical nature of the condition; b) the gaps in knowledge, skills, attitudes, and resources of PWD/caregivers and their primary caregivers, thus affecting the active engagement of the latter; and c) the broader systemic and structural barriers negatively affecting the context of dementia care. As previously outlined, from the methods reported in this systematic review, a significant part requires long-term training (i.e., 4–12 weeks) and could not be accessible to a vast parcel of elderlies outside metropolitan areas or modest resource centers (72). Thus, one of the significant challenges is the home-setting adaptation of well-established double-blind, placebo-controlled protocols. In this scenario, both PwD and care providers should be encouraged to influence the organization and living environment of care homes whenever possible (13). Also, some evidence has highlighted the role of ethnicity and cultural background (e.g., Hispanic and Afro-Americans) and the importance of religious coping (73) in the context of psychosocial intervention and recommend the inclusion of ethnic and cultural variables in a more comprehensive program (74, 75). Gender differences, particularly in symptom profile, living condition, and coping style and response, seem to affect the outcome of psychosocial intervention, as highlighted by the literature (76). Another relevant aspect is the educational attainment of PwD (77); interestingly, prior evidence has suggested more significant benefits of cognitive intervention among higher educated patients (77). The importance of continuous follow-up, support, and professional reinforcement, mostly offering help based on the family’s needs, has been outlined in previous studies with no benefit of psychosocial interventional (35, 78).

The present work has some limitations that deserve further comment. First, the broad scope of the theme, encompassing studies with multiple methods and outcomes. Second, the difficulty in transposing the current evidence to the real scenario of the COVID-19 pandemics, particularly in different continents and socio-cultural and economic realities. Although there is a vast multiplicity of psychosocial health programs for the old age with dementia and behavioral disturbances, we expect to bring a sum of the well-succeeded initiatives and, through that, global insights directed to best practices of caring for this population. We believe that future programs targeting behavioral disturbances and caregiver mental health issues in dementia shall consider general principles such as those briefly commented in our review.

## Concluding Remarks

Before the COVID-19 epidemy, many studies have invested in evidence-based models targeting the provision of personalized interventions to implement community-based customized dementia care. Conversely, the experience of dealing with social isolation during the pandemic period will demand research on preventive and protective factors of dementia and the pursue of efficient intervention from every perspective, notably the domestic setting. The summary of the evidence from the last ten years suggests that low-cost techniques, tailored to the dyad, with increasing use of technology through friendly online platforms and application robots, can counteract the team’s physical absence during the COVID-19 Pandemic. Such techniques should be directed to mood, sleep, and physical exercise, exploring playful music and dance activities. The potential benefits of different programs are substantial: Improve mood in dementia, reduce lack of mobility, decrease social isolation and integrate the outcomes with more general medical support, helping avoid complications and early recognition of delirium and other physical problems. Also, the caregiver’s self-monitoring, the further understanding of the PwD symptoms, the development of a sense of competence, well-being, and the treatment of mood changes in caregivers are crucial endpoints. Other aspects still to explore are related to adapting the protocols to distant areas or where the pandemics have increased. The integration of support networks with expertise centers is also essential. Lastly, it is also essential to acknowledge the importance of real-world studies, even when limited by resources and strict bias control. Therefore, the existing studies may provide useful information on the effect size of specific interventions, the optimal number of sessions, participants enrolled in staff supervision in different scenarios. The future investigation, supporting the implementation of evidence-based psychosocial interventions, will help optimizing training programs for caregivers in post-pandemic times.

## Author Contributions

GA: method design, systematic review of literature and results compilation (including creation of figures and tables), writing of the manuscript (abstract, introduction, methods, results, discussion and conclusions), selection and organization of bibliographic references. MD: method design, systematic review of literature and results compilation (including creation of figures and tables), writing of the manuscript (abstract, introduction, methods, results, discussion and conclusions), selection and organization of bibliographic references. VR: discussion of the theory and method, critical review and text editing. MC: discussion of the theory, critical review and text editing. AV: discussion of the theory and method, writing of the manuscript, critical review and text editing. CC: discussion of theory, writing of the manuscript and critical review. EB: discussion of the theory and method, writing of the manuscript, critical review and text editing.

## Conflict of Interest

The authors declare that the research was conducted in the absence of any commercial or financial relationships that could be construed as a potential conflict of interest.

## Glossary

**Table:**

BASE	Behavior Analytics & Support Enhancement
PwD	Patients with dementia
BPSD	Behavior and Psychological Symptoms of Dementia
CG	Control Group
DANCE	Dance Therapy Intervention
DMAS-17	Dementia Mood Assessment Scale
WHELD	Well-being and Health for People with Dementia
BASE	Behavior Analytics & Support Enhancement
BPSD	Behavioral and Psychological Symptoms of Dementia
NPI‐NH	Neuropsychiatric Inventory—Nursing Home version
SMQ	Short‐Memory Questionnaire
ATC	Anatomical Therapeutic Chemical classification
HASK	Hearing Aid Skills and Knowledge test
SENSE-CogGSKTV	SENSE-Cog Glasses Skills and Knowledge Test for Vision
SENSE-Cog FA	SENSECog Functional Assessment
BGSI	Bangor Goal Setting Inventory
SENSE-Cog CM	SENSE-Cog Communications Manual
DEMQOL	Dementia Quality of Life
BADL	Bristol Activities of Daily Living scale
VALV-VFQ	Veterans Affairs Low Vision-Visual Functioning Questionnaire
VALV-VFQS	Veterans Affairs Low Vision-Visual Functioning Questionnaire Spousal rating
HHIE	Hearing Handicap Inventory for the Elderly
HHIES	Hearing Handicap Inventory for the Elderly Spousal rating
NPI	Neuropsychiatric Inventory
RSS	Relationship Satisfaction Scale
12-GHQ	12-item General Health Questionnaire
12-SFHS	Short Form-12 Health Survey
FCR	Family Caregiving Role scale
HAD	Hospital Anxiety and Depression scale
RUD-L	RUD-Lite instrument; EQ-5D-5L, 5-level EuroQol 5-dimension
NPI-NH	Neuropsychiatric Inventory – Nursing Home version
SCIDS	Sense of Competence in Dementia Care Staff
RMBPC	Revised Memory and Behavior Problems Checklist
CBI	Caregiver Burden Inventory
WCCL-R	Revised Ways of Coping Checklist
MBI	Mindfulness-based Interventions
CSDD	Cornell Scale for Depression in Dementia
RAID	Rating Anxiety in Dementia Scale
QLAD	Quality of Life Alzheimer’s Disease scale
MMSE	Mini Mental State Examination
PSS-13	Perceived Stress Scale
MBAS	Meditation Breath Attention Scores
CMAI-SF	14-item Cohen-Mansfield Agitation Inventory Short Form
RUDAS	Rowland Universal Dementia Assessment Scale
PPQ	e Program Participation Questionnaire
SSCQ	Short Sense of Competence Questionnaire
MSPSS	Multidimensional Scale of Perceived Support
SSL12-I	Social Support List 12-Interactions
LS	Loneliness Scale
LSNS-6	Lubben Social Network Scale
ICECAP-O	e Investigating Choice Experiments for the Preferences of Older People Capability Measure for Older People
CarerQol	Care Related Quality of Life scale
CRA	Caregiver Reaction Assessment
DEM-DISC	Dementia Digital Interactive Social Chart
MDS	Minimum Dataset
CANE	Camberwell Assessment of Needs for the Elderly
QoL-AD	13-item Quality of Life – AD
EQ5D+c	Health-Related Quality of Life extended with a cognitive dimension
USE	Usefulness, ease of use, ease of learning and satisfaction questionnaire
CDR-SOB	Clinical Dementia Rating Scale Sum of Boxes
CERAD-NB	Consortium to Establish a Registry for Alzheimer’s Disease neuropsychological battery
ADCS-ADL	Alzheimer’s Disease Cooperative Study-Activities of Daily Living Inventory
VAS	Visual Analog Scale; BDI, 21-item Beck Depression Inventory
SOC	29-item sense of coherence scale
HRQoL	The generic health-related quality of life instrument (15D)
BVC	Brøset Violence Checklist
NPI-Q	Neuropsychiatric Inventory– Questionnaire
GBS-scale	The Gottfries-Bråne-Steen scale
ADL	Katz Index of Independence in Activities of Daily Living
SSCQ	The Short Sense of Competence Questionnaire
PSS	The Perceived Stress Scale
PMS	The Pearlin Mastery Scale
CES-D	Center for Epidemiological Studies Depression Scale
HADS-A	Hospital Anxiety and Depression Scale
CDR	Clinical Dementia Rating Scale
MBP	Memory and behavioral problems
Ilfeld short version	Ilfeld Psychiatric Symptoms Index
NYUCI-AC	New York University Caregiver Intervention-Adult Child
GDS	Geriatric Depression Scale
RMBPC	24-item Revised Memory and Behavior Problems Checklist
START	STrAtegies for RelaTives
CMAI	The Cohen-Mansfield Agitation Inventory
EQ-VAS	Self-rated quality of life
CSDD	Cornell Scale for Depression in Dementia
ACE-R	Addenbrooke’s Cognitive Examination-Revised
RUD	Resource Utilisation In Dementia Questionnaire
EQ-5D	EuroQoL 5-dimensions
QALY	Quality-Adjusted Life-Year
FITT-NH	Family Intervention: Telephone Tracking-Nursing Home
ZBI	Zarit Burden Interview
NH Hassles	Nursing Home Hassles Scale
RMBC	Revised Memory and Behavior Checklist
FITT-C	Telephone Tracking –Caregiver
FAD	Family Assessment Device
SEQ	Self-Efficacy Questionnaire
PAC	Positive Aspects of Caregiving
CDR	Clinical Dementia Rating
IADL	Instrumental Activities of Daily Living
APID	Appropriate Psychotropic drugs use In Dementia index
NPI-Q	Neuropsychiatric Inventory Questionnaire
CANE	Camberwell Assessment of Need for the Elderly
UBOS	Utrecht Burnout Scale
ADK	Alzheimer’s Disease Knowledge test
SF-36	Short-Form Health Survey
SSES	Self-Efficacy Scale
iCST	individual cognitive stimulation therapy
TAU	Treatment As Usual
ADAS-Cog	Alzheimer’s Disease Assessment Scale–Cognitive Subscale
QoL-AD	Quality of Life Alzheimer Disease Scale
BADLS	Bristol Activities of Daily Living Scale
QCPR	Quality of the Carer–Patient Relationship Scale
SF-12	Short Form-12 Health Survey
RS-14	Resilience Scale
HDRS	Hamilton Depression Rating Scale
TMAS	Taylor Manifest Anxiety Scale
DRKQ	Dementia Related Knowledge Questionnaire
BAI	Beck Anxiety Inventory
WFCS	The Work–Family Conflict Scale
RRS	Ruminative Responses Scale
PSMS	Physical Self-Maintenance Scale
ARTEMIS	ART Encounters: Museum Intervention Study
CODEM	Communication Behavior In People With Dementia
OERS	Observed Emotion Rating Scale
NPI-NH	Neuropsychiatric Inventory – Nursing Home Version
MBSR	Mindfulness-based Stress Reduction
SS	Social Support
FAST	Functional Assessment Staging of Alzheimer’s Disease
TICS	Telephone Interview for Cognitive Status
AAQ II	10-item Acceptance and Action Questionnaire II
POMS	Profile of Mood States
MOSSF-36	Medical Outcomes Study Short-Form Health Survey
FCIMS	15-item Mutuality scale of the Family Care Inventory.
