# Supplementary material for: A Systematic Review of Home-Setting Psychoeducation Interventions for Behavioral Changes in Dementia: Some Lessons for the COVID-19 Pandemic and Post-Pandemic Assistance
Source: Front Psychiatry. 2020 Sep 29;11:577871. doi: 10.3389/fpsyt.2020.577871 (PMC7550734; doi:10.3389/fpsyt.2020.577871)
Supplement: Supplementary file 1 [file DataSheet_1.pdf]

Table 1. Characteristics of cohort studies with patients with dementia and caregivers[§]

Authors, Year	SELECTION	COMPARABILITY	OUTCOME
Arritxabal et al., 2011 (1)	****	**	**
Bartels et al., 2019 (2)	****	*	***
Boersma et al., 2017 (3)	***	*	**
Brown et al., 2016 (4)	****	**	***
Bruvik et al., 2013 (5)	****	**	***
Burns et al., 2010 (6)	****	**	***
Chen et al., 2015 (7)	****	**	***
Clarke et al., 2017 (8)	****	**	***
Dam et al., 2017 (9)	****	**	***
Davis et al., 2011 (10)	****	**	***
Den IJssel et al., 2019 (11)	****	**	***
Fossey et al., 2019 (12)	****	**	***
Gaugler et al., 2018 (13)	****	*	***
Guzmán et al., 2016 (14)	**	**	**
Johannessen et al., 2014 (15)	****	**	***
Johannessen et al., 2015 (16)	****	**	***
Koivisto et al., 2016 (17)	****	**	***
Langhammer et al., 2019 (18)	***	*	**
Liang et al., 2017 (19)	****	**	***
Lord et al., 2017 (20)	****	**	***
Matsuzomo et al., 2015 (21)	****	**	***
McCurry et al., 2011 (22)	****	**	***
McCurry et al., 2012 (23)	****	**	***
Moyle et al., 2019 (24)	****	**	***
Nakanishi et al., 2017 (25)	****	**	***
Nakanishi et al., 2018 (26)	****	**	***
Orrell et al., 2017 (27)	****	*	***
Phung et al., 2013 (28)	****	**	***
Pihet et al., 2018 (29)	***	*	**
Shata et al., 2017 (30)	****	**	***
Søgaard et al., 2014a (31)	****	**	***

Søgaard et al., 2014b (32)	****	**	***
Tremont et al., 2013 (33)	****	**	***
Tremont et al., 2016 (34)	****	**	***
Van Mierlo et al., 2015 (36)	****	**	***

§§*NewCastle-Ottawa quality assessment scale for cohort studies*. The item selection (range 0-4) refers to the representativeness of the exposed cohort, selection of the non-exposed cohort, ascertainment of exposure, and whether the outcome of interest was present at the start of the study. The item comparability (range 1 -2) refers to cohorts' comparability based on the design or analysis. The item outcome (range 0-3) comprises the items outcome assessment, the duration, and adequacy of follow-up measurement.

Table 2. Characteristics of case-control studies with patients with dementia and caregivers§§

Authors, Year	SELECTION	COMPARABILITY	EXPOSURE
Dahlrup et al., 2011 (37)	****	**	***
Gaugler et al., 2011 (38)	****	**	***
Jones et al., 2018 (39)	**	**	**
Karel et al. 2016 (40)	**	**	**
Kerssens et al., 2015 (41)	**	**	**
Stockwell-Smith et al., 2018 (42)	****	**	***
Williams et al., 2010 (43)	****	**	***

§§*NewCastle-Ottawa quality assessment scale for case control studies*. The item selection (range 0-4) and refers to the adequacy of case definition, representativeness of the cases, selection and definition of controls. The item comparability ranges (range 1-2) and refers to the comparability of cases and controls based on the design or analysis. The item exposure (ranges 0-3) comprises the items ascertainment of exposure, the same method of ascertainment for cases and controls, and non-response rate.

REFERENCES

1. Etxeberria Arritxabal I, García Soler A, Iglesias Da Silva A, Urdaneta Artola E, Lorea González I, Díaz Veiga P, et al. [Effects of training in emotional regulation strategies on the well-being of carers of Alzheimer patients]. Rev Esp Geriatr Gerontol. 2011 Aug;46(4):206–12.

2. Bartels SL, van Knippenberg RJM, Köhler S, Ponds RW, Myin-Germeys I, Verhey FRJ, et al. The necessity for sustainable intervention effects: lessons-learned from an experience sampling intervention for spousal carers of people with dementia. Aging Ment Health. 2019 Aug 1;1–11.

3. Boersma P, van Weert JCM, van Meijel B, Dröes R-M. Implementation of the Veder contact method in daily nursing home care for people with dementia: a process analysis according to the RE-AIM framework. J Clin Nurs. 2017 Feb;26(3–4):436–55.

4. Brown KW, Coogle CL, Wegelin J. A pilot randomized controlled trial of mindfulness-based stress reduction for caregivers of family members with dementia. Aging Ment Health. 2016;20(11):1157–66.

5. Bruvik FK, Allore HG, Ranhoff AH, Engedal K. The effect of psychosocial support intervention on depression in patients with dementia and their family caregivers: an assessor-blinded randomized controlled trial. Dement Geriatr Cogn Dis Extra. 2013;3(1):386–97.

6. Burns A, Mittelman M, Cole C, Morris J, Winter J, Page S, et al. Transcultural influences in dementia care: observations from a psychosocial intervention study. Dement Geriatr Cogn Disord. 2010;30(5):417–23.

7. Chen H-M, Huang M-F, Yeh Y-C, Huang W-H, Chen C-S. Effectiveness of coping strategies intervention on caregiver burden among caregivers of elderly patients with dementia. Psychogeriatrics. 2015 Mar;15(1):20–5.

8. Churcher Clarke A, Chan JMY, Stott J, Royan L, Spector A. An adapted mindfulness intervention for people with dementia in care homes: feasibility pilot study. Int J Geriatr Psychiatry. 2017;32(12):e123–31.

9. Dam AEH, de Vugt ME, van Boxtel MPJ, Verhey FRJ. Effectiveness of an online social support intervention for caregivers of people with dementia: the study protocol of a randomised controlled trial. Trials. 2017 29;18(1):395.

10. Davis JD, Tremont G, Bishop DS, Fortinsky RH. A telephone-delivered psychosocial intervention improves dementia caregiver adjustment following nursing home placement. Int J Geriatr Psychiatry. 2011 Apr;26(4):380–7.

11. van Duinen-van den IJssel JCL, Bakker C, Smalbrugge M, Zwijsen SA, Appelhof B, Teerenstra S, et al. Effects on staff outcomes from an intervention for management of neuropsychiatric symptoms in residents of young-onset dementia care units: A cluster randomised controlled trial. Int J Nurs Stud. 2019 Aug;96:35–43.

12. Fossey J, Garrod L, Tolbol Froiland C, Ballard C, Lawrence V, Testad I. What influences the sustainability of an effective psychosocial intervention for people with dementia living in care homes? A 9 to 12-month follow-up of the perceptions of staff in care homes involved in the WHELD randomised controlled trail. Int J Geriatr Psychiatry. 2019;34(5):674–82.

13. Gaugler JE, Reese M, Mittelman MS. The Effects of a Comprehensive Psychosocial Intervention on Secondary Stressors and Social Support for Adult Child Caregivers of Persons With Dementia. Innov Aging. 2018 Jun;2(2):igy015.

14. Guzmán A, Freeston M, Rochester L, Hughes JC, James IA. Psychomotor Dance Therapy Intervention (DANCIN) for people with dementia in care homes: a multiple-baseline single-case study. Int Psychogeriatr. 2016;28(10):1695–715.

15. Johannessen A, Povlsen L, Bruvik F, Ulstein I. Implementation of a multicomponent psychosocial programme for persons with dementia and their families in Norwegian municipalities: experiences from the perspective of healthcare professionals who performed the intervention. Scand J Caring Sci. 2014 Dec;28(4):749–56.

16. Johannessen A, Bruvik FK, Hauge S. Family carers' experiences of attending a multicomponent psychosocial intervention program for carers and persons with dementia. J Multidiscip Healthc. 2015;8:91–9.

17. Koivisto AM, Hallikainen I, Välimäki T, Hongisto K, Hiltunen A, Karppi P, et al. Early psychosocial intervention does not delay institutionalization in persons with mild Alzheimer disease and has impact on neither disease progression nor caregivers' well-being: ALSOVA 3-year follow-up. Int J Geriatr Psychiatry. 2016 Mar;31(3):273–83.

18. Langhammer B, Sagbakken M, Kvaal K, Ulstein I, Nåden D, Rognstad MK. Music Therapy and Physical Activity to Ease Anxiety, Restlessness, Irritability, and Aggression in Individuals With Dementia With Signs of Frontotemporal Lobe Degeneration. J Psychosoc Nurs Ment Health Serv. 2019 May 1;57(5):29–37.

19. Liang A, Piroth I, Robinson H, MacDonald B, Fisher M, Nater UM, et al. A Pilot Randomized Trial of a Companion Robot for People With Dementia Living in the Community. J Am Med Dir Assoc. 2017 Oct

1;18(10):871–8.

20. Lord K, Rapaport P, Cooper C, Livingston G. Disseminating START: training clinical psychologists and admiral nurses as trainers in a psychosocial intervention for carers of people with dementia's depressive and anxiety symptoms. BMJ Open. 2017 Aug 21;7(8):e017759.

21. Matsuzono K, Yamashita T, Ohta Y, Hishikawa N, Sato K, Kono S, et al. Clinical Benefits for Older Alzheimer's Disease Patients: Okayama Late Dementia Study (OLDS). Journal of Alzheimer's Disease [Internet]. 2015 Jun 25 [cited 2017 May 10];46(3):687–93. Available from: http://www.medra.org/servlet/aliasResolver?alias=iospress&doi=10.3233/JAD-150175

22. McCurry SM, Pike KC, Vitiello MV, Logsdon RG, Larson EB, Teri L. Increasing Walking and Bright Light Exposure to Improve Sleep in Community-Dwelling Persons with Alzheimer's Disease: Results of a Randomized, Controlled Trial: WALKING AND LIGHT TO IMPROVE SLEEP IN AD. Journal of the American Geriatrics Society [Internet]. 2011 Aug [cited 2020 Jun 14];59(8):1393–402. Available from: http://doi.wiley.com/10.1111/j.1532-5415.2011.03519.x

23. McCurry SM, LaFazia DM, Pike KC, Logsdon RG, Teri L. Development and evaluation of a sleep education program for older adults with dementia living in adult family homes. Am J Geriatr Psychiatry. 2012 Jun;20(6):494–504.

24. Moyle W, Murfield J, Jones C, Beattie E, Draper B, Ownsworth T. Can lifelike baby dolls reduce symptoms of anxiety, agitation, or aggression for people with dementia in long-term care? Findings from a pilot randomised controlled trial. Aging Ment Health. 2019;23(10):1442–50.

25. Nakanishi M, Endo K, Hirooka K, Granvik E, Minthon L, Nägga K, et al. Psychosocial behaviour management programme for home-dwelling people with dementia: A cluster-randomized controlled trial. Int J Geriatr Psychiatry. 2018;33(3):495–503.

26. Nakanishi M, Hirooka K, Imai Y, Inoue S, Yukari Y, Katayama C, et al. Dementia Care Competence Among Care Professionals and Reduced Challenging Behavior of Home-Dwelling Persons with Dementia: A Pre- and Post-Intervention Data Analysis. J Alzheimers Dis. 2018;64(2):515–23.

27. Orrell M, Yates L, Leung P, Kang S, Hoare Z, Whitaker C, et al. The impact of individual Cognitive Stimulation Therapy (iCST) on cognition, quality of life, caregiver health, and family relationships in dementia: A randomised controlled trial. Brayne C, editor. PLOS Medicine [Internet]. 2017 Mar 28 [cited 2020 Jun 14];14(3):e1002269. Available from: https://dx.plos.org/10.1371/journal.pmed.1002269

28. Phung KTT, Waldorff FB, Buss DV, Eckermann A, Keiding N, Rishøj S, et al. A three-year follow-up on the efficacy of psychosocial interventions for patients with mild dementia and their caregivers: the multicentre, rater-blinded, randomised Danish Alzheimer Intervention Study (DAISY). BMJ Open. 2013 Nov 21;3(11):e003584.

29. Pihet S, Kipfer S. Coping with dementia caregiving: a mixed-methods study on feasibility and benefits of a psycho-educative group program. BMC Geriatr. 2018 10;18(1):209.

30. Shata ZN, Amin MR, El-Kady HM, Abu-Nazel MW. Efficacy of a multi-component psychosocial intervention program for caregivers of persons living with neurocognitive disorders, Alexandria, Egypt: A randomized controlled trial. Avicenna J Med. 2017 Jun;7(2):54–63.

31. Søgaard R, Sørensen J, Waldorff FB, Eckermann A, Buss DV, Waldemar G. Cost analysis of early psychosocial intervention in Alzheimer's disease. Dement Geriatr Cogn Disord. 2014;37(3–4):141–53.

32. Søgaard R, Sørensen J, Waldorff FB, Eckermann A, Buss DV, Phung KTT, et al. Early psychosocial intervention in Alzheimer's disease: cost utility evaluation alongside the Danish Alzheimer's Intervention Study (DAISY). BMJ Open. 2014 Jan 15;4(1):e004105.

33. Tremont G, Davis J, Papandonatos GD, Grover C, Ott BR, Fortinsky RH, et al. A telephone intervention for dementia caregivers: background, design, and baseline characteristics. Contemp Clin Trials. 2013 Nov;36(2):338–47.

34. Tremont G, Davis JD, Ott BR, Galioto R, Crook C, Papandonatos GD, et al. Randomized Trial of the Family Intervention: Telephone Tracking-Caregiver for Dementia Caregivers: Use of Community and Healthcare Resources. Journal of the American Geriatrics Society [Internet]. 2017 May [cited 2020 Jun 19];65(5):924–30. Available from: http://doi.wiley.com/10.1111/jgs.14684

35. Tremont G, Davis JD, Bishop DS, Fortinsky RH. Telephone-Delivered Psychosocial Intervention Reduces Burden in Dementia Caregivers. Dementia (London). 2008;7(4):503–20.

36. Van Mierlo LD, Meiland FJM, Van de Ven PM, Van Hout HPJ, Dröes R-M. Evaluation of DEM-DISC, customized e-advice on health and social support services for informal carers and case managers of people with dementia; a cluster randomized trial. Int Psychogeriatr. 2015 Aug;27(8):1365–78.

37. Dahlrup B, Nordell E, Andrén S, Elmståhl S. Family caregivers' assessment of symptoms in persons with dementia using the GBS-scale: differences in rating after psychosocial intervention--an 18-month follow-up study. Clin Interv Aging. 2011;6:9–18.

38. Gaugler JE, Roth DL, Haley WE, Mittelman MS. Modeling trajectories and transitions: results from the New York University caregiver intervention. Nurs Res. 2011 Jun;60(3 Suppl):S28-37.

39. Jones C, Moyle W, Murfield J, Draper B, Shum D, Beattie E, et al. Does Cognitive Impairment and Agitation in Dementia Influence Intervention Effectiveness? Findings From a Cluster-Randomized-Controlled Trial With the Therapeutic Robot, PARO. J Am Med Dir Assoc. 2018;19(7):623–6.

40. Karel MJ, Teri L, McConnell E, Visnic S, Karlin BE. Effectiveness of Expanded Implementation of STAR-VA for Managing Dementia-Related Behaviors Among Veterans. Gerontologist. 2016 Feb;56(1):126–34.

41. Kerssens C, Kumar R, Adams AE, Knott CC, Matalenas L, Sanford JA, et al. Personalized technology to support older adults with and without cognitive impairment living at home. Am J Alzheimers Dis Other Demen. 2015 Feb;30(1):85–97.

42. Stockwell-Smith G, Moyle W, Kellett U. The impact of early psychosocial intervention on self-efficacy of care recipient/carer dyads living with early-stage dementia-A mixed-methods study. J Adv Nurs. 2018 May 13;

43. Williams VP, Bishop-Fitzpatrick L, Lane JD, Gwyther LP, Ballard EL, Vendittelli AP, et al. Video-based coping skills to reduce health risk and improve psychological and physical well-being in Alzheimer's disease family caregivers. Psychosom Med. 2010;72(9):897–904.
