# Supplementary material for: A Systematic Review of Home-Setting Psychoeducation Interventions for Behavioral Changes in Dementia: Some Lessons for the COVID-19 Pandemic and Post-Pandemic Assistance
Source: Front Psychiatry. 2020 Sep 29;11:577871. doi: 10.3389/fpsyt.2020.577871 (PMC7550734; doi:10.3389/fpsyt.2020.577871)
Supplement: Supplementary file 2 [file DataSheet_2.docx]

Figure 1 – Studies flow chart

**Records identified through database searching – PUBMED-Medline, ISI, Embase (n = 588)
(n =65)**

Full-text articles excluded, lack of follow-up measurements (n =4)

## Included

**Records after duplicates removed
(n = 580)**

Records excluded on basis of title and abstract
(n = 373)

## Eligibility

## Screening

## Identification

Studies included in qualitative synthesis
(n = 43)

Records screened on basis of title and abstract (n=207)

(n
(n =39)

Full-text articles assessed for eligibility
(n = 92)

**Additional records identified through other sources
(n = 35 )**
